# Comprehensive and Quantitative Analysis of the Changes in Proteomic and Phosphoproteomic Profiles during Stimulation and Repression of Steroidogenesis in MA-10 Leydig Cells †

**DOI:** 10.3390/ijms232112846

**Published:** 2022-10-25

**Authors:** Zoheir B. Demmouche, Jacques J. Tremblay

**Affiliations:** 1 Reproduction, Mother and Child Health, Room T3-67, CHU de Québec—Université Laval Research Centre, Québec, QC G1V 4G2, Canada; 2 Centre for Research in Reproduction, Development and Intergenerational Health, Department of Obstetrics, Gynecology and Reproduction, Faculty of Medicine, Université Laval, Québec, QC G1V 0A6, Canada

**Keywords:** testis, Leydig cells, steroidogenesis, star, AMPK, phosphoproteomics, proteomics

## Abstract

Leydig cells produce testosterone, a hormone essential for male sex differentiation and spermatogenesis. The pituitary hormone, LH, stimulates testosterone production in Leydig cells by increasing the intracellular cAMP levels, which leads to the activation of various kinases and transcription factors, ultimately stimulating the expression of the genes involved in steroidogenesis. The second messenger, cAMP, is subsequently degraded to AMP, and the increase in the intracellular AMP levels activates AMP-dependent protein kinase (AMPK). Activated AMPK potently represses steroidogenesis. Despite the key roles played by the various stimulatory and inhibitory kinases, the proteins phosphorylated by these kinases during steroidogenesis remain poorly characterized. In the present study, we have used a quantitative LC-MS/MS approach, using total and phosphopeptide-enriched proteins to identify the global changes that occur in the proteome and phosphoproteome of MA-10 Leydig cells during both the stimulatory phase (Fsk/cAMP treatment) and inhibitory phase (AICAR-mediated activation of AMPK) of steroidogenesis. The phosphorylation levels of several proteins, including some never before described in Leydig cells, were significantly altered during the stimulation and inhibition of steroidogenesis. Our data also provide new key insights into the finely tuned and dynamic processes that ensure adequate steroid hormone production.

## 1. Introduction

Leydig cells are located in the interstitial space between the seminiferous tubules of the mammalian testis [1,2]. These cells are the source of androgens, the main one being testosterone [3]. Steroidogenesis is the biological process of converting cholesterol into steroid hormones, which involves cholesterol transport into the mitochondria, where steroidogenesis is initiated. In males, androgens are essential for male sex differentiation during fetal life and for initiating and maintaining spermatogenesis from puberty onwards. Androgens are also needed to acquire male secondary sex characteristics during puberty. Inadequate androgen production is associated with some cases of differences/disorders of sex development (DSD) in males [4].

Since androgens have pleiotropic roles in male reproductive function and overall health, their synthesis is regulated tightly. Steroidogenesis in Leydig cells is stimulated mainly by the pituitary luteinizing hormone (LH) [5]. The binding of LH to its G protein-coupled receptor (LHCGR) on Leydig cells activates adenylate cyclase, which leads to an increase in intracellular cAMP levels [6]. LH/cAMP-induced steroidogenesis then triggers the activation of several kinases, including protein kinase A (PKA), mitogen-activated protein kinase (MAPK), and calcium/calmodulin-dependent protein kinase I (CAMKI), which in turn phosphorylates several proteins required for increased steroid hormone synthesis (reviewed in [7]). This includes several transcription factors, such as the cAMP response element-binding protein (CREB)/CRE modulator (CREM), GATA4, and SF1 (reviewed in [8]). Furthermore, the de novo synthesis of the NR4A1/NUR77 (nuclear receptor subfamily 4, group A, member 1) transcription factor is required for maximal hormone-induced steroidogenesis [9]. The induction of *Nr4a1* expression and the stimulation of steroidogenesis in Leydig cells also requires the release of Ca^2+^ from internal stores through the ryanodine receptors, leading to the activation of CAMKI [10]. Once adequate testosterone levels are reached, stimulation of steroidogenesis is blunted by two mechanisms. The first is the classic negative feedback loop, where testosterone acts at the level of the hypothalamus and pituitary to inhibit LH production [11]. The second is at the level of the Leydig cell itself. In Leydig cells, cAMP is degraded into AMP by phosphodiesterase (PDE) 8A, PDE8B, and PDE4 [12]. The ensuing increase in the intracellular AMP levels activates AMP-activated protein kinase (AMPK), a ubiquitous serine/threonine kinase best known as an energy balance sensor. AMPK is a heterotrimeric complex containing one catalytic subunit (alpha) and two regulatory subunits (beta and gamma) [13]. Once activated, AMPK potently blunts the LH/cAMP-induced steroidogenesis in Leydig cells [14].

We previously compared the transcriptome of Leydig cells that were either untreated, stimulated with Forskolin (Fsk, an agonist of adenylate cyclase), or co-treated with Fsk+AICAR (an agonist of AMPK). This led to the identification and characterization of several genes that were upregulated by the LH/cAMP stimulatory pathway and subsequently downregulated by the AMPK repressive pathway [14]. In addition to the changes in the transcriptome, the protein levels are also affected by the stimulation/repression of steroidogenesis in Leydig cells. However, global changes in protein and phosphoprotein levels have never been reported in this context. In the present work, we have used a quantitative mass spectrometry approach to elucidate the global and dynamic changes in the phosphoproteome of Leydig cells in response to stimulatory (Fsk/cAMP) and inhibitory (AICAR/AMPK) treatments.

## 2. Results

### 2.1. Validation of MA-10 Leydig Cell Responsiveness

**Figure 1:**
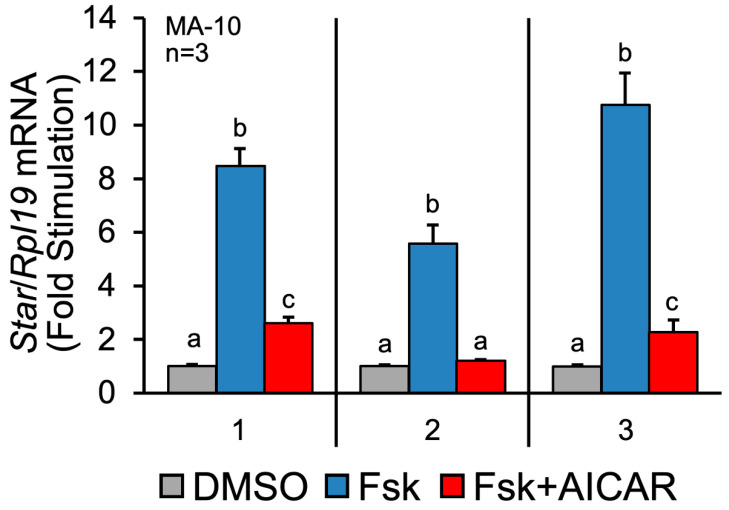
Validation of treatments on MA-10 Leydig cells by assessing *Star* mRNA levels. MA-10 Leydig cells were treated with either DMSO (control, grey bars), Forskolin alone (Fsk, 10 µM, blue bars) or Forskolin and AICAR (Fsk+AICAR, 10 µM, and 1 mM, respectively, red bars) for 1 h. Total RNA was extracted and reverse-transcribed, and qPCR was performed to quantify *Star* mRNA levels. *Rpl19* was used to normalize the data. The numbers on the x-axis refer to the three different samples used in the LC-MS/MS analysis. Results are displayed as the mean of three individual experiments, each performed in duplicate. For a given experiment, different letters indicate a statistically significant difference between groups (*p* < 0.05).

Before analyzing the samples by quantitative LC-MS/MS, we first validated the responsiveness of the MA-10 Leydig cells to the different treatments. MA-10 Leydig cells were treated for 1 h with the vehicle (DMSO), Forskolin alone (Fsk), or Fsk+AICAR. Fsk is an agonist of adenylate cyclase, leading to increased intracellular cAMP levels and stimulation of steroidogenesis. AICAR is an agonist of the AMPK kinase, which we have identified as a potent repressor of hormone-activated steroidogenesis [14]. Although 1 h is sufficient to detect changes in the protein phosphorylation levels, it is usually too short to detect changes in the total protein levels by Western blot. We, therefore, isolated total RNA, which was used in a qPCR to quantify the mRNA levels for the steroidogenic acute regulatory (*Star*) protein. The *Star* gene codes for the steroidogenic acute regulatory (STAR) protein, a protein essential for hormone-induced cholesterol transport into mitochondria and, consequently, steroidogenesis [15]. The *Star* gene is an excellent marker of the dynamic steroidogenic process in Leydig cells, as its expression is strongly induced by LH/Fsk/cAMP and repressed by AICAR/AMPK ([14] and reviewed in [8]). As shown in Figure 1, the *Star* mRNA levels from the three samples used in LC-MS/MS (described below) were increased six- to ten-fold in the presence of Fsk. As expected [14], this increase was potently repressed when the MA-10 Leydig cells were co-treated with AICAR in addition to Fsk (Figure 1). These results confirm the responsiveness of the MA-10 Leydig cells to Fsk and AICAR and validate that the protein samples isolated from these cells are suitable for LC-MS/MS analysis.

### 2.2. Treatment of MA-10 Leydig Cells with Fsk or Fsk+AICAR Significantly Affects the Levels of 20 Proteins

Total proteins from the MA-10 Leydig cells treated for 1 h with either the vehicle (DMSO), Fsk alone, or Fsk+AICAR were extracted, digested, and quantitively analyzed by LC-MS/MS. A total of 5887 proteins were identified, and 4819 were quantified (data not shown). The level of the majority of these proteins was not significantly changed by the treatments due to the short treatment time. Only 20 proteins (listed in Table 1) were significantly affected between treatments (DMSO vs. Fsk, Fsk vs. Fsk+AICAR, and DMSO vs. Fsk+AICAR). The protein levels either increased (shown in red in Table 1) or decreased (shown in blue in Table 1). In addition, the same protein was sometimes found in different comparison groups. For instance, the protein levels of the orphan nuclear receptor NR4A1 (NUR77), a known regulator of the hormone-induced steroidogenic gene expression in Leydig cells [10,16], was increased by 2.4-fold by the Fsk treatment (DMSO vs. Fsk group), and this increase was blunted after the AICAR treatment (down by 2.34-fold; Fsk vs. Fsk+AICAR group), as previously reported [14]. Since the levels of the NR4A1 protein are back down to the control levels after treatment with Fsk+AICAR, the NR4A1 protein levels were not significantly changed in the DMSO vs. Fsk+AICAR group. These changes in NR4A1 protein levels (increased by Fsk/cAMP and reduced upon activation of AMPK by AICAR) serve as a positive control and validate the quantitative LC-MS/MS approach used.

The level of the protein Dymeclin, a Golgi-associated protein believed to be involved in intracellular trafficking [17,18,19], was the most increased after 1 h of Fsk treatment (23-fold; Table 1). Similar to NR4A1, the Fsk-dependent increase of Dymeclin was prevented when AMPK was activated by AICAR (decreased by 23-fold and back to control levels). Another protein affected by both Fsk and AICAR is the Mpv17-like protein 2 (MPV17L2). MPV17L2 is required for the assembly and stability of the mitochondrial ribosome, and in its absence, protein synthesis in mitochondria is impaired, and mitochondrial DNA aggregates [20]. Contrary to NR4A1 and Dymeclin, the MPV17L2 levels were reduced by 5.35-fold in the presence of Fsk and increased by 5.23-fold by AICAR-mediated AMPK activation (Table 1).

As summarized in Table 1, in addition to Dymeclin (up by 23-fold) and NR4A1 (up by 2.4-fold), the top Fsk-induced proteins included the homologous-pairing protein 2 homolog (PSMC3IP; up by 5-fold), acyl-coenzyme A dehydrogenase family member 12 (ACAD12; up by 4-fold), and N-acetyltransferase domain containing 1 (NATD1; up by 3.6-fold). In addition to MPV17L2, the most induced proteins in the presence of AICAR (activation of AMPK) included the cyclic AMP-responsive element-binding protein 1 (CREB1; up by 18-fold), while decreased proteins, in addition to Dymeclin and NR4A1, included the Lemur serine/threonine-protein kinase 2 (LMTK2; down by 2.8-fold). These results showed that after a short 1-h treatment, the level of some proteins involved in mitochondrial function and in steroidogenesis was already altered.

### 2.3. Identification of Proteins Differentially Phosphorylated in MA-10 Leydig Cells in Response to Fsk and AICAR

Our quantitative analysis of the phosphoproteome of MA-10 Leydig cells revealed significant changes in the phosphorylation levels of several phosphopeptides corresponding to various proteins in response to the treatments. The treatments included DMSO (the vehicle used as the control), Fsk to increase the cAMP levels mimicking LH-stimulated steroidogenesis, and Fsk+AICAR simultaneously, where AICAR activates AMPK, a kinase previously reported to potently inhibit hormone-induced steroidogenesis [14]. The top 10 differentially phosphorylated (up and down) phosphopeptides for each pairwise comparison (Fsk vs. DMSO, Fsk+AICAR vs. Fsk, Fsk+AICAR vs. DMSO) are presented in Table 2.

#### 2.3.1. Proteins Differentially Phosphorylated in Response to Fsk

**Figure 2:**
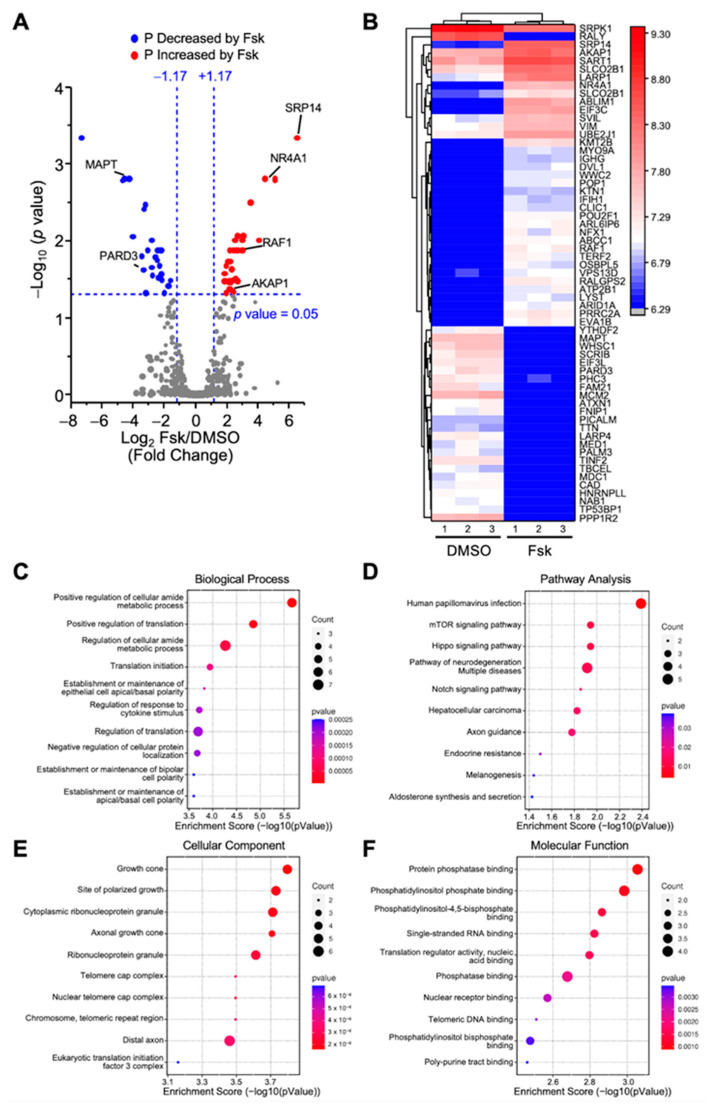
Analysis of differentially phosphorylated proteins in MA-10 Leydig cells in response Forskolin. LC-MS/MS was performed on enriched phosphopeptides extracted and digested from MA-10 Leydig cells treated with either DMSO (control) or Forskolin (Fsk, 10 µm) for 1 h. Phosphorylated proteins were then quantified and compared between treatments. Results are the mean of three individual experiments, each performed in triplicate. (**A**) Volcano plot comparing the level of phosphorylated proteins detected by LC-MS/MS in control and Fsk-treated MA-10 Leydig cells. Each dot represents a distinct phosphoprotein. A Log2 fold change of +/−1.17 (corresponds to 2.25-fold) with a *p*-value of 0.05 was used and is represented by the blue dotted lines. In response to Fsk, blue dots depict a decrease in phosphorylation level (two proteins are identified in that group: MAPT and PARD3), while red dots correspond to an increase in phosphorylation level (four proteins are identified in that group: SRP14, NR4A1, RAF1, AKAP1). Grey dots correspond to proteins with no significant changes in phosphorylation levels. (**B**) Heatmap of protein phosphorylation levels for differentially phosphorylated proteins between treatment groups (control DMSO vs. Fsk). The name of the protein is indicated. The scale on the right represents expression levels (Log10). Biological Process (**C**), Pathway Analysis (**D**), Cellular Component (**E**), and Molecular Function (**F**) of the differentially phosphorylated proteins are shown.

We first compared the phosphopeptides between the control and Fsk-treated MA-10 Leydig cells to identify changes in protein phosphorylation during the stimulatory phase of steroidogenesis. This led to the identification of 12,125 phosphopeptides, of which 8471 were quantified. As shown in Figure 2A, the quantified phosphopeptides were mapped on a volcano plot based on the significance and the ratio between the Fsk-treated and control (DMSO) MA-10 Leydig cells. A total of 61 phosphopeptides had significantly different phosphorylation levels between these two conditions; the phosphorylation of 35 phosphopeptides was increased, while the phosphorylation of 26 phosphopeptides was decreased following the Fsk treatment. These 61 phosphopeptides correspond to 60 unique phosphoproteins and are shown along with their relative phosphorylation levels between the two conditions on the heatmap in Figure 2B.

The proteins exhibiting increased phosphorylation after the Fsk treatment included the nuclear receptor NR4A1, the signal recognition particle SRP14, the protein kinase A anchoring protein AKAP1, and the serine/threonine kinase RAF1. In comparison, the proteins showing a reduction in their phosphorylation level in response to Fsk included the microtubule-associated protein tau (MAPT) and the Par-3 family cell polarity regulator protein PARD3 (Figure 2A,B). In response to Fsk in MA-10 Leydig cells, the differentially phosphorylated proteins are mostly implicated in the metabolic processes and the regulation of translation (Figure 2C). A pathway analysis revealed that these proteins are implicated mainly in the mTOR and Hippo signaling pathways, two pathways involved in metabolism (Figure 2D). As shown in Figure 2E, the differentially phosphorylated proteins are associated with various cellular components. Finally, the 60 differentially phosphorylated proteins are associated with various molecular functions, including binding to protein phosphatase, phosphatidyl inositol phosphate, and nucleic acid (Figure 2F).

#### 2.3.2. Proteins Differentially Phosphorylated in Response to AMPK Activation

**Figure 3:**
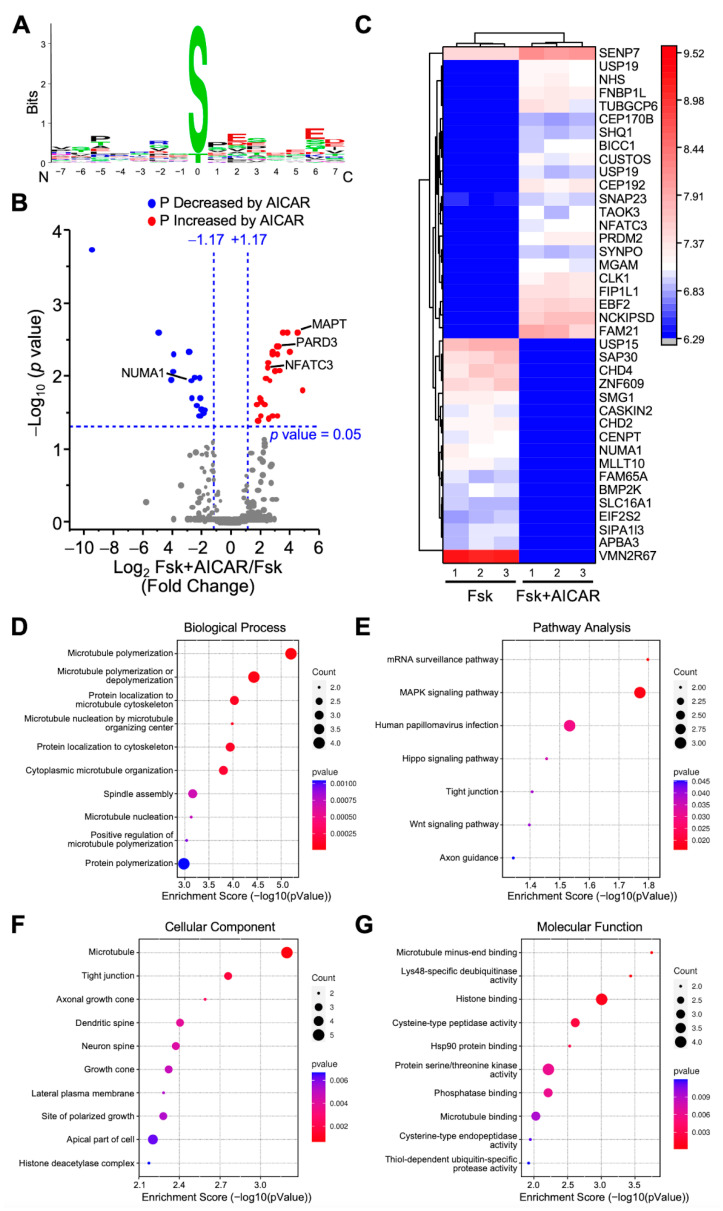
Analysis of differentially phosphorylated proteins in MA-10 Leydig cells treated with Forskolin or Forskolin+AICAR. LC-MS/MS was performed on enriched phosphopeptides extracted and digested from MA-10 Leydig cells treated with Forskolin (Fsk, 10 µm) or Fsk+AICAR (10 µM and 1 mM, respectively) for 1 h. Phosphorylated proteins were then quantified and compared between treatments. Results are the mean of three individual experiments, each performed in triplicate. (**A**) Consensus sequence motif of phosphorylated peptides. The amino acid chosen is the one with the highest probability of being phosphorylated. (**B**) Volcano plot comparing the level of phosphorylated proteins detected by LC-MS/MS in Fsk-treated vs. Fsk+AICAR-treated MA-10 Leydig cells (to observe the effects of AICAR). Each dot represents a distinct phosphoprotein. A Log2 fold change of +/−1.17 (corresponds to 2.25-fold) with a *p*-value of 0.05 was used and is represented by the blue dotted lines. In response to AICAR, blue dots depict a decrease in phosphorylation level (one protein is identified in that group: NUMA1), while red dots correspond to an increase in phosphorylation level (three proteins are identified in that group: MAPT, PARD3, NFATC3). Grey dots correspond to proteins with no significant changes in phosphorylation levels. (**C**) Heatmap of protein phosphorylation levels for differentially phosphorylated proteins between treatments (Fsk vs. Fsk+AICAR). The name of the protein in indicated. The scale on the right represents expression levels (Log10). Biological Process (**D**), Pathway Analysis (**E**), Cellular Component (**F**), and Molecular Function (**G**) of the differentially phosphorylated proteins are shown.

The activation of AMPK with AICAR represses LH/cAMP-induced steroidogenesis but does not significantly affect the unstimulated steroidogenic cells [14]. Therefore, to identify potential target proteins of AMPK, we compared all phosphopeptides between the Fsk- and Fsk+AICAR-treated MA-10 Leydig cells. The differences between those two conditions should reflect mainly the proteins phosphorylated by AMPK. This comparison identified 12,125 phosphopeptides, of which 8581 phosphopeptides were quantified. It also revealed that the phosphorylated amino acid in cells exposed to AICAR was, by far, a serine, as shown by the sequence motif presented in Figure 3A. Quantified phosphopeptides were then mapped on a volcano plot based on the significance and the ratio between the Fsk+AICAR- and Fsk-treated cells (Figure 3B). A total of 46 phosphopeptides had significantly different phosphorylation levels between these two conditions; in the Fsk+AICAR group, the phosphorylation of 27 phosphopeptides was increased, while the phosphorylation of 19 phosphopeptides was decreased compared to the Fsk group (Figure 3B,C). These 46 phosphopeptides correspond to 44 unique phosphoproteins, as some proteins have more than one phosphorylated residue (phosphopeptide sequence). These 44 phosphoproteins are presented along with their relative phosphorylation levels between the two conditions on the heatmap presented in Figure 3C.

The proteins with increased phosphorylation in the AICAR group included NFATC3 (transcription factor), PARD3, and MAPT; an example of a protein with reduced phosphorylation in the AICAR group is the nuclear mitotic apparatus protein NUMA1 (Figure 3A,B). The proteins differentially phosphorylated between the Fsk+AICAR and Fsk groups are mostly implicated in the microtubule polymerization or its organization (Figure 3D). This is supported by the localization of these proteins, mostly with microtubules (Figure 3F), with their main molecular functions being microtubule binding, histone binding, and deubiquitinase activity (Figure 3G). The pathway analysis revealed that the 44 proteins are mainly associated with the MAPK signaling pathway and mRNA surveillance pathway (Figure 3E).

#### 2.3.3. Proteins Differentially Phosphorylated in Response to Combined Fsk+AICAR

**Figure 4:**
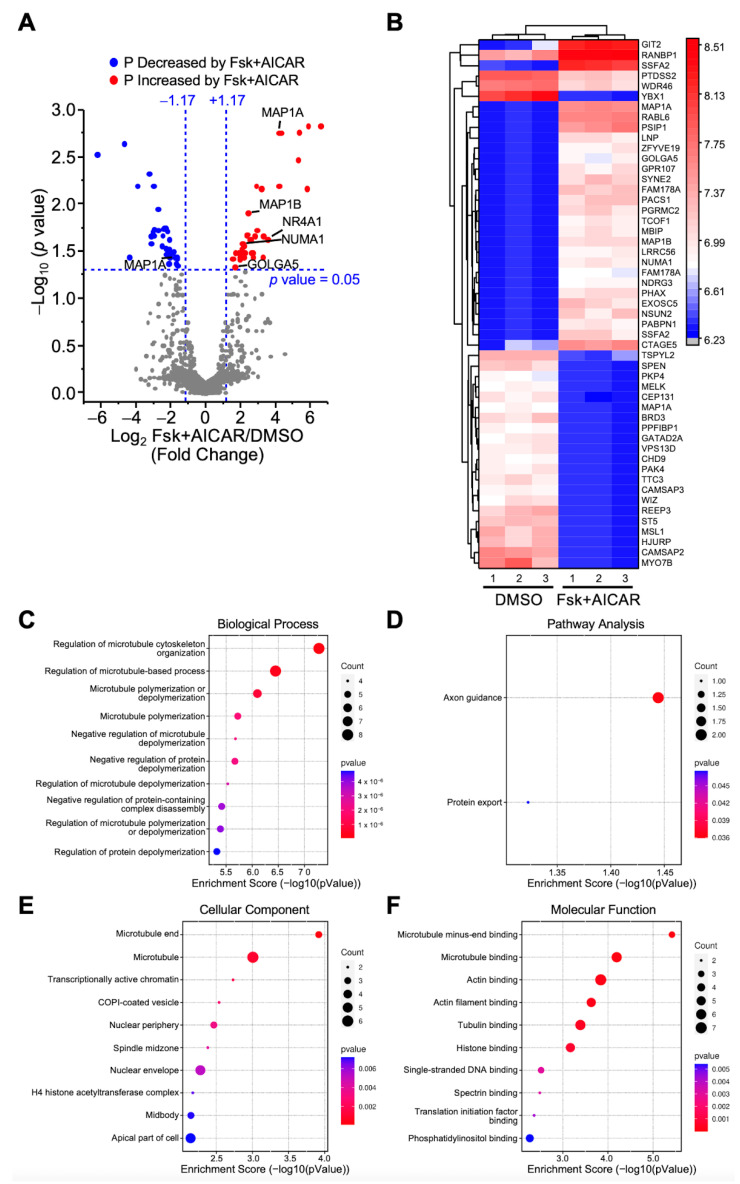
Analysis of differentially phosphorylated proteins in MA-10 Leydig cells in response to Forskolin+AICAR. LC-MS/MS was performed on enriched phosphopeptides extracted and digested from MA-10 Leydig cells treated with DMSO (control) or Fsk+AICAR (10 µM and 1 mM, respectively) for 1 h. Phosphorylated proteins were then quantified and compared between treatments. Results are the mean of three individual experiments, each performed in triplicate. (**A**) Volcano plot comparing the level of phosphorylated proteins detected by LC-MS/MS in control (DMSO) vs. Fsk+AICAR-treated MA-10 Leydig cells. Each dot represents a distinct phosphoprotein. A Log2 fold change of +/−1.17 (corresponds to 2.25-fold) with a *p*-value of 0.05 was used and is represented by the blue dotted lines. In response to Fsk+AICAR, blue dots depict a decrease in phosphorylation level (one protein is identified in that group: MAP1A), while red dots correspond to an increase in phosphorylation level (five proteins are identified in that group: MAP1A, MAP1B, NR4A1, NUMA1, GOLGA5). Grey dots correspond to proteins with no significant changes in phosphorylation levels. (**B**) Heatmap of protein phosphorylation levels for differentially phosphorylated proteins between treatments (control DMSO vs. Fsk+AICAR). The name of the protein is indicated. The scale on the right represents expression levels (Log10). Biological Process (**C**), Pathway Analysis (**D**), Cellular Component (**E**) and Molecular Function (**F**) of the differentially phosphorylated proteins are shown.

Comparisons were made between the Fsk+AICAR and the control (DMSO) groups to identify all the changes in the protein phosphorylation triggered by the simultaneous treatment of the MA-10 Leydig cells with Fsk (stimulatory) and AICAR (inhibitory). A total of 12,125 phosphopeptides were identified, of which 8711 were quantified. As shown in Figure 4A, the quantified phosphopeptides were mapped on a volcano plot based on the significance and the ratio between the Fsk+AICAR and control (DMSO) cells. A total of 73 phosphopeptides exhibited significantly different phosphorylation levels between these two conditions; the phosphorylation of 38 phosphopeptides was increased in the MA-10 Leydig cells treated with Fsk+AICAR, while the phosphorylation of 35 phosphopeptides was decreased. These 73 phosphopeptides correspond to 68 unique phosphoproteins, as some proteins harbor more than one phosphorylation site, such as MAP1A. The 68 phosphoproteins identified, along with their relative phosphorylation levels between the two conditions, are shown on the heatmap presented in Figure 4B.

Some of the proteins with increased phosphorylation in the MA-10 Leydig cells in response to Fsk+AICAR included NR4A1, NUMA1, the protein GOLGA5 involved in maintaining the Golgi structure, and the microtubule-associated proteins MAP1A and MAP1B. MAP1A was also present amongst the proteins with reduced phosphorylation. This is explained by the fact that MAP1A contains two distinct phosphopeptides: one showed increased phosphorylation, while the other showed reduced phosphorylation in the presence of Fsk+AICAR (Figure 4A). In terms of the biological processes, differentially phosphorylated proteins were mainly associated with microtubule polymerization, depolymerization, and organization (Figure 4C). Consistent with this, these proteins are mostly localized with microtubules, and their main functions are to bind to the microtubules, actin, and tubulin (Figure 4F). The pathway analysis associated the differentially phosphorylated proteins with the axon guidance pathway (Figure 4D).

Taken together, these results provide the first description of the global changes in the phosphoproteome of MA-10 steroidogenic Leydig cells after a stimulatory treatment (Fsk) and an inhibitory treatment (activation of AMPK by AICAR).

### 2.4. Phosphorylation Trajectory of Representative Proteins

Several proteins exhibited an altered phosphorylation status in response to Fsk and to Fsk+AICAR in the MA-10 Leydig cells (Figure 2, Figure 3 and Figure 4). Although the protein levels remained unchanged for the majority of proteins (Table 1), changes in the phosphorylation level in response to Fsk and to Fsk+AICAR could still be the result of a concomitant change in the protein level. Therefore, the phosphorylation status of the representative proteins was plotted and compared to their protein levels, allowing for better visualization of the phosphorylation trajectory of a given protein.

**Figure 5:**
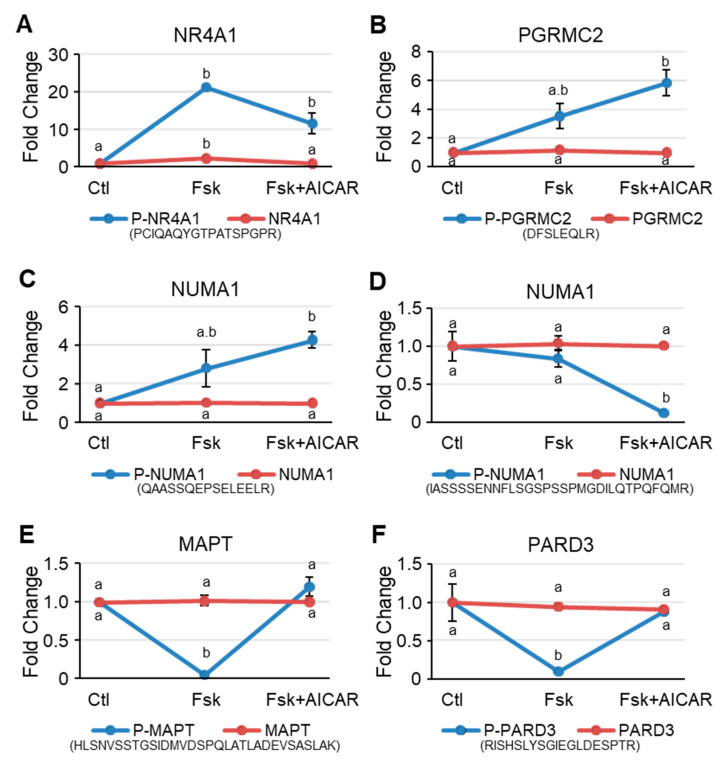
Representative proteins that are differentially phosphorylated in MA-10 Leydig cells in response to Forskolin and to Forskolin+AICAR. Intensity plots of representative phosphoproteins (blue lines) and total proteins (red lines). Ctl, DMSO-treated cells; Fsk, Forskolin-treated cells; Fsk+AICAR, cells treated with Forskolin and AICAR. Results are shown as Fold Change compared to the control. Results are the mean of three individual experiments +/−SEM. The phosphopeptide sequence is shown beneath the graph. For a given protein, different letters indicate a statistically significant difference between treatment groups (*p* < 0.05).

The phosphorylation level of NR4A1, an orphan nuclear receptor known to regulate the expression of several steroidogenic genes, was increased by more than 20-fold after the Fsk treatment, while the total NR4A1 protein level was only increased by 2.4-fold. This increase tended to be attenuated when cells were treated with AICAR in addition to Fsk (Figure 5A). Interestingly, the phosphorylation levels of the progesterone membrane receptor components 2 (PGRMC2), a protein belonging to the membrane-associated progesterone receptor (MAPR) protein family, was significantly increased when cells were treated with Fsk and with Fsk+AICAR (Figure 5B). No change in the total PGRMC2 protein level was noted. The NUMA1 exhibited the same phosphorylation pattern as PGRMC2 (Figure 5C), with an increase in phosphorylation of the phosphopeptide QAASSQEPSELEELR. On the other hand, a different phosphopeptide (IASSSSENNFLSGSPSSPMGDILQTPQFQMR) of the NUMA1 protein remained unchanged in response to Fsk, whereas AICAR caused a significant reduction in its phosphorylation level (Figure 5D). Finally, the phosphorylation level of two proteins, known to be downstream of AMPK, MAPT, and PARD3, was significantly reduced when the MA-10 Leydig cells were treated with Fsk. However, this decrease in phosphorylation was no longer apparent when the cells were co-treated with Fsk+AICAR, suggesting that AMPK phosphorylates these proteins (Figure 5E,F). The total protein level of PGRMC2, NUMA1, MAPT, and PARD3 was not affected by the treatments. These results validate AMPK activation in the MA-10 Leydig cells treated with AICAR.

Altogether, these results reveal that protein phosphorylation, in response to Fsk and Fsk+AICAR in MA-10 Leydig cells, is a very dynamic process and is mainly independent of the changes in protein levels.

## 3. Discussion

Several studies have reported changes in the expression of a single gene or its protein levels in response to hormone-induced steroidogenesis. However, Leydig cell steroidogenesis is a strictly regulated process that requires changes in gene expression, protein levels, and protein phosphorylation of many genes and proteins. Therefore, the main objective of this work was to elucidate the global phosphoproteomic changes that occur in MA-10 Leydig cells in response to Forskolin-induced steroidogenesis, followed by AICAR-activated AMPK, leading to repression of steroidogenesis.

### 3.1. Global Variation in Protein Levels in MA-10 Leydig Cells 1 h after Fsk or Fsk+AICAR Treatment

We first determined the changes in the global protein levels and found that the level of only 20 proteins was significantly modified in the Leydig cells treated for 1 h with either Forskolin alone (stimulatory) or Forskolin+AICAR (inhibitory). One of those proteins was the nuclear receptor NR4A1. Our global proteomic analysis revealed that the NR4A1 protein levels were increased by 2.4-fold after the Forskolin treatment, and this increase was blunted when cells were co-treated with AICAR. This is consistent with previous studies from our group that reported the pattern of *Nr4a1* mRNA levels [14], as well as a time course of NR4A1 protein levels [10], in response to (Bu)_2_-cAMP treatment of mouse MA-10 Leydig cells and primary Leydig cells from rats. Therefore, the observed changes in the NR4A1 protein levels in our current study validate our experimental approach.

Another protein affected by the treatments was Dymeclin, which was induced 23-fold in response to Fsk and then lost upon AMPK activation. Although it has never been reported in Leydig cells, Dymeclin is believed to be implicated in vesicle trafficking, as it shuttles between the cytosol and the Golgi apparatus in a highly dynamic manner [18,19]. Vesicle trafficking is an important process for steroidogenesis. For instance, the life cycle of lipid droplets, which are a source of cholesterol substrate for steroid hormones [21], involves intracellular trafficking (reviewed in [22]). The significant and rapid changes in Dymeclin protein levels in response to Fsk and Fsk+AICAR suggest that Dymeclin might play an active role in hormone-regulated steroidogenesis in Leydig cells.

The transcription factor, CREB, is known to activate steroidogenic gene expression in Leydig cells [23,24,25,26,27,28]. Despite this, the CREB protein levels remained unchanged in response to the Fsk/cAMP treatment, which is identical to what we observed in our current global proteomic analysis. Unexpectedly, the CREB protein levels were increased by 18-fold in the MA-10 Leydig cells treated with AICAR to turn on the repressive kinase, AMPK (Fsk+AICAR-treated MA-10 Leydig cells compared to the Fsk-treated cells). This would appear to suggest a repressive role for CREB in steroidogenesis. Although mainly known as an activator, CREB has also been reported to act as a repressor. For instance, CREB represses the expression of the *cFos* gene [29]. Furthermore, alternate exon usage is also known to switch CREB from an activator to a repressor [30]. It is, therefore, possible that activated AMPK influences CREB exon usage and that CREB would, therefore, repress steroidogenic gene expression in that context.

The MPV17L2 protein levels were also modulated by the treatments. MPV17L2 is an integral mitochondrial inner membrane protein [20], and as such, it is implicated in mitochondrial ribosome assembly [20] and in reactive oxygen species (ROS) production [31], two processes known to influence steroidogenesis in Leydig cells [32]. We found that the MPV17L2 protein levels increased 5-fold in the presence of AICAR, while it was reduced by 5-fold in the Fsk-treated cells. Our data are consistent with transcriptomic data from the pancreas, where the *Mpv17l2* mRNA levels are decreased by 1.6-fold in AMPK-deficient mice [33], indicating that activated AMPK increases its expression.

Overall, our data show that globally, the levels of 20 proteins, including some never before reported in Leydig cells, are rapidly and differentially modulated in response to Fsk (stimulatory) and to Fsk+AICAR (inhibitory).

### 3.2. Comprehensive Analysis of the Phosphoproteome during Stimulation and Inhibition of Steroidogenesis in MA-10 Leydig Cells

In Leydig cells, the response to hormone stimulation involves the activation of various signaling pathways, leading to increased activity of various kinases, including PKA, MAPK, CAMKI, and AMPK. These kinases then phosphorylate various proteins, thus ensuring a proper cellular response to the stimulus. Although some phosphorylated proteins have been identified, the majority of studies have focused on a single protein at the time. One study reported the changes in the phosphoproteome of Leydig cells in which the cAMP levels were maintained artificially high by inhibiting all phosphodiesterase activity [34]. This study identified alterations in several phosphosites, some of which were also detected in our current study. However, constitutively high cAMP levels might trigger non-physiological responses. In our present work, we used Fsk, an agonist of adenylate cyclase that leads to a typical increase in cAMP levels, triggering a stimulatory response, and AICAR, an agonist of AMPK, leading to an inhibitory response. In both cases, the levels of cAMP, the main second messenger in Leydig cells, followed a normal increase–decrease rate typical of a more classic response [14]. Using this treatment scheme and a quantitative phosphoproteomics approach, we now report the global changes in the phosphoproteome that occur in Leydig cells in response to both a stimulatory and an inhibitory signal. We found that the phosphorylation level of a large number of proteins was modified in response to either Fsk (stimulatory response) or Fsk+AICAR (inhibitory response).

The proteins displaying increased phosphorylation levels in the MA-10 Leydig cells treated with Fsk include AKAP1 (also known as AKAP121), RAF1, NR4A1, and SRP14. The phosphorylation of both AKAP1 and RAF1 was previously reported as being altered in the MA-10 Leydig cells depleted of PDE4 and PDE8 activity [34]. PDE activity is required for normal Leydig cell function. Indeed, PDEs regulate steroidogenesis by their ability to degrade cAMP into AMP, thus inactivating several cAMP-dependent pathways and kinases, such as PKA, while simultaneously activating the repressive kinase AMPK [12]. AKAP1 is a member of the AKAP family of scaffold proteins. AKAP1 is known to interact with PKA and therefore regulates its intracellular localization, especially at the outer mitochondrial membrane (OMM) (reviewed in [35]), where PKA can phosphorylate its target proteins [36]. A key target of PKA at the OMM for the stimulation of steroidogenesis is the STAR protein [36,37]. PKA-dependent phosphorylation of STAR increases its cholesterol shuttling activity, leading to enhanced steroidogenesis [36,37]. AKAP1 is, therefore, considered an important mitochondrial signaling hub, and the mitochondrion is an essential organelle for steroidogenesis in Leydig cells. To date, AKAP1 is not known to be phosphorylated. Our current work demonstrates that AKAP1 is phosphorylated in response to Fsk. The physiological implication of AKAP1 phosphorylation remains to be established, although a previous study suggested that phosphorylation of AKAP1 can modulate the AKAP1-PKA association [38].

Another protein exhibiting increased phosphorylation in response to Fsk is SRP14. SRP14 is involved in protein translation and, more specifically, in the elongation arrest and efficient translocation of proteins into the endoplasmic reticulum [39]. This is consistent with the requirement of a de novo protein synthesis in hormone-induced steroidogenic cells [10]. Interestingly, the level of SRP14 protein in glioblastoma cells is increased by CYP17A1 [40], an enzyme implicated in steroidogenesis. As we observed in the Fsk-treated MA-10 Leydig cells, SRP14 was also found to be phosphorylated during a large screen of phosphorylated proteins in Ras-transformed cells [41]. The significance of SRP14 phosphorylation, however, remains unknown.

The proteins displaying reduced phosphorylation levels after Fsk treatment include MAPT and PARD3. Phosphorylation of both proteins was restored when AMPK was activated (Fsk+AICAR-treated cells). MAPT, a protein involved in microtubule assembly and stabilization, is a known target of AMPK [42]. Furthermore, phosphorylation of MAPT is reduced in the MA-10 Leydig cells deficient in PDE4 and PDE8 activity to maintain high cAMP levels [34]. These data are consistent with our present data showing a reduction in MAPT phosphorylation in the context of high cAMP levels (Fsk-treated cells) and increased phosphorylation in AMPK-activated cells. Phosphorylation of MAPT is known to influence protein stability and degradation (reviewed in [43]).

The protein PARD3 is part of the PAR3/PAR6/aPKC complex implicated in tight-junction assembly [44]. Activation of AMPK facilitates tight-junction assembly [45]. Whether activated AMPK interacts with and directly phosphorylates the PAR3/PAR6/aPKC complex remains uncertain [45]. However, our current data now indicate that PARD3 is phosphorylated upon AMPK activation. Furthermore, PARD3 is known to be phosphorylated by the PAR1 kinase, leading to PARD3 translocation from the tight junction to the cytosol [46]. Once in the cytosol, PARD3 promotes the interaction between PP1A phosphatase and LATS1, a mediator of the Hippo signaling pathway [46]. This results in the dephosphorylation and inactivation of LATS1 and, consequently, dephosphorylation, activation, and nuclear translocation of the TAZ transcriptional co-activator [46]. Once in the nucleus, TAZ represses Leydig cell steroidogenesis by suppressing the expression of several steroidogenic genes via an interaction with the nuclear receptor NR4A1, preventing it from binding to DNA and activating its target genes [47]. It is tempting to speculate that the phosphorylation of PARD3, which we observed in the AMPK-activated Leydig cells, might lead to the same events, therefore contributing to a repression of steroidogenesis.

The transcription factors belonging to the nuclear factor of activated T cells (NFAT) family are well-known transcriptional activators (reviewed in [48,49]). In our phosphoproteomic screen, the phosphorylation of the transcription factor NFATC3 remained unchanged in the presence of Fsk alone. However, it was significantly increased in the MA-10 Leydig cells treated with Fsk+AICAR, indicating that NFATC3 becomes phosphorylated upon activation of AMPK. Interestingly, phosphorylated NFAT transcription factors are known to be sequestered in the cytoplasm since phosphorylation masks the nuclear localization sequence [50,51]. The phosphorylation of NFATC3 that occurs upon activation of AMPK in MA-10 Leydig cells would be consistent with the exclusion of NFATC3 from the nucleus and a reduction in its ability to activate gene expression, therefore contributing to reduced steroidogenesis.

Another group of differentially phosphorylated proteins includes those where the phosphorylation level was significantly different only in the Fsk+AICAR-treated MA-10 Leydig cells vs. control cells (vehicle-treated). This included the NUMA1 protein, which was differentially phosphorylated on two different peptides; one exhibited increased phosphorylation, while the phosphorylation of another residue of the protein was significantly decreased. NUMA1 is a component of the nuclear matrix (reviewed in [52]) and is implicated in ciliogenesis and autophagy, as is AMPK [53]. NUMA1 was indeed proposed as a potential target of AMPK, although this remains to be validated [54]. Our current results support direct phosphorylation of NUMA1 by AMPK.

The PGRMC2 protein also exhibited increased phosphorylation in the MA-10 Leydig cells treated with Fsk+AICAR vs. controls. PGRMC2 is a membrane-associated progesterone receptor (MAPR) present in Leydig cells and is believed to play a role in the progesterone auto/paracrine action on Leydig cells [55,56] in addition to potentially regulating the activity of some cytochrome P450 enzymes [57]. We showed that PGRMC2 is present in MA-10 Leydig cells and that it is phosphorylated after AMPK activation.

The MA-10 Leydig cell line is the gold standard for studying Leydig cells, although they are not identical to the cultured primary Leydig cells. The MA-10 Leydig cell line was originally established from a Leydig cell tumor in a mouse (M5480P) [58] and has since been meticulously characterized. Treatment of MA-10 cells with the luteinizing hormone, Forskolin, or cAMP analogs, increases steroid hormone production in the same way as in normal Leydig cells ([58] and reviewed in [59]). The rate-limiting step in hormone-induced steroidogenesis is the transport of cholesterol into the mitochondria, where steroidogenesis is initiated (reviewed in [60]); this is also true for MA-10 Leydig cells [10,15,61]. MA-10 cells mainly produce progesterone because of a mutation in the *Cyp17a1* coding sequence [58]. MA-10 Leydig cells are, therefore, suitable for the study of hormone-induced steroidogenesis and, more specifically, the early steps of steroidogenesis [62]. Since our current work focuses on the modulation of protein phosphorylation that occurs early following hormone treatment, the MA-10 Leydig cell line is a suitable and convenient model to use.

In conclusion, using a quantitative approach, our present study determined the global phosphoproteomic profile of MA-10 Leydig cells in response to Fsk (stimulation of steroidogenesis) and AICAR-mediated AMPK activation (repression of steroidogenesis). Our results indicate that steroidogenesis is a very dynamic process that involves differential protein phosphorylation by various kinases, altogether contributing to the fine-tuned regulation that is needed to achieve the appropriate production of steroid hormones.

## 4. Materials and Methods

### 4.1. Cell Culture

The MA-10 cell line was obtained from ATCC (Cat# CRL-3050, RRID:CVCL_D789. ATCC, Manassas, VA, USA). The MA-10 cells were grown in a DMEM/F12 medium supplemented with 2.438 g/L sodium bicarbonate, 3.57 g/L HEPES, and 15% horse serum on gelatin-coated plates. Penicillin and streptomycin sulphate were added to the cell culture media to a final concentration of 50 mg/L, and the cells were kept at 37 °C, 5% CO_2_, in a humidified incubator. The MA-10 Leydig cell line was validated by morphology and by quantifying the progesterone output, as previously described.

### 4.2. Chemicals

The AMPK agonist, AICAR, was obtained from Tocris Bioscience (Minneapolis, MN, USA). Forskolin (Fsk) was purchased from Sigma-Aldrich, Canada (Oakville, ON, Canada).

### 4.3. RNA Isolation, Reverse Transcription, and Quantitative PCR

The MA-10 Leydig cells were cultured in the presence of either DMSO (vehicle), Fsk (10 µM), or Fsk+AICAR (10 µM + 1 mM) for 60 min. Isolation of RNA, cDNA synthesis, and reverse transcription-quantitative PCR (RT-qPCR) were performed as previously described [63,64]. Briefly, the total RNA from the MA-10 Leydig cells grown and treated, as described above, was isolated using TRIZOL (Life Technologies, Burlington, ON, Canada) and reverse-transcribed using the iScript Advanced cDNA Synthesis Kit (Bio-Rad Laboratories, ON, Canada). A quantitative real-time PCR was performed using a CFX96™ Real-Time PCR Detection System (Bio-Rad Laboratories, ON, Canada) along with the SsoAdvanced Universal SYBR Green Supermix kit (Bio-Rad Laboratories, ON, Canada) according to the manufacturer’s protocols. Relative expression was normalized to the expression of *Rpl19*, which was used as an internal control. The mouse *Star* primers were: forward 5′-GTT CCT CGC TAC GTT CAA GC-3′ and reverse 5′-GAA ACA CCT TGC CCA CAT CT-3′. The mouse *Rpl19* primers were: forward 5′-CTG AAG GTC AAA GGG AAT GTG-3′ and reverse 5′-GGA CAG AGT CTT GAT GAT CTC-3′. The melting temperature of both primer couples was 62.6 °C.

### 4.4. Sample Preparation for LC-MS/MS

The MA-10 Leydig cells (5.8 million) were plated in 15-cm plates and then treated at 80% confluence with either the vehicle (DMSO), Fsk (10 μM) or Fsk+AICAR (10 µM + 1 mM) for 60 min at 37 °C and 5% CO_2_. This experiment was repeated three times in triplicate.

Protein extraction, digestion, phosphopeptide enrichment, and mass spectrometry analyses were performed by the proteomics platform of the CHU de Quebec Research Centre (Quebec City, QC, Canada). Cell pellets were resuspended in an extraction buffer (50 mM ammonium bicarbonate, 0.5% sodium deoxycholate (SDC), 50 mM DTT, protease inhibitor cocktail, phosphatase inhibitor PhosSTOP, and 1 μM pepstatin) and vortexed for 1 min. They were then sonicated with a microprobe (Sonic Dismembrator 550, Fisher Scientific) 20 times for 1 s (on/off). The extract was centrifuged at 16,000× *g* for 15 min, the supernatant was collected, and then acetone-precipitated. The pellet was resuspended in ammonium bicarbonate 50 mM/SDC 1% and treated with bioruptor 15 times for 30 s (on/off). The protein concentration was determined by performing a Bradford assay.

Protein aliquots of 10 μg for the global protein analysis or 2 mg for the phosphopeptide analysis for each condition (vehicle, Fsk, Fsk+AICAR) were used. The proteins were first heated at 95 °C for 5 min and reduced with 0.2 mM DTT at 37 °C 30 min. They were then alkylated with 0.8 mM IAA (iodoacetamide) for 30 min at 37 °C in the dark. This was followed by digestion with trypsin (1:50) at 37 °C overnight. Digestion was stopped with the addition of formic acid; the sodium deoxycholate was removed after centrifugation. The peptides were desalted using a stage-tip column (for the global protein analysis) or HLB column (for the phosphopeptide analysis).

Phosphopeptide enrichment was performed using the High Select Phosphopeptide Enrichment Kits & Reagents kit (Fisher Scientific Canada, Catalog number A32993) according to the manufacturer’s protocol. Two (2) mg of dry protein from each condition (vehicle, Fsk, Fsk+AICAR) were resuspended in 150 μL of binding/equilibration buffer (pH < 3). The TiO_2_ spin-tip columns were washed and then equilibrated before adding 150 μL of the resuspended samples. The TiO_2_ spin-tip columns loaded with the samples were centrifuged twice at 1000× *g* for 5 min to bind the phosphopeptides. The TiO_2_ spin-tip columns were then washed twice with the binding/equilibration buffer, once with the wash buffer and then with HPLC water. The phosphopeptides were finally eluted with 50 μL of phosphopeptide elution buffer twice.

### 4.5. Quantitative Sample Analysis by LC-MS/MS

Aliquots of 1 μg (for global protein analysis) and 1 mg of enriched phosphopeptide were analyzed by nanoLC-MS/MS using a Dionex UltiMate 3000 nanoRSLC chromatography system (Thermo Fisher Scientific, Ontario, Canada), connected to an Orbitrap Fusion mass spectrometer (Thermo Fisher Scientific Ontario, Canada). Peptides were trapped at 20 μL/min in a loading solvent (2% acetonitrile, 0.05% TFA) on a 5 mm × 300 μm C18 Pepmap cartridge pre-column (Thermo Fisher Scientific, Ontario, Canada) for 5 min. The pre-column was then switched online with a Pepmap Acclaim column (Thermo Fisher Scientific, Ontario, Canada) 50 cm × 75 μm internal diameter separation column. The peptides were eluted with a linear gradient of 5–40% solvent B (A: 0.1% formic acid, B: 80% acetonitrile, 0.1% formic acid) over 270 min for a total of a 300-min run at 300 nL/min for the global protein analysis. For the phosphopeptide analysis, elution with the solvent gradient was completed in 90 min for a total run of 120 min. Mass spectra were acquired using a data-dependent acquisition mode using Thermo Xcalibur software, version 4.1.50. Full-scan mass spectra (350 to 1800 m/z) were acquired in the orbitrap using an AGC target of 4e5, a maximum injection time of 50 ms, and a resolution of 120,000. An internal calibration using lock mass on the m/z 445.12003 siloxane ion was used. Each MS scan was followed by the acquisition of fragmentation involving MS/MS spectra of the most intense ions for a total cycle time of 3 s (top speed mode). The selected ions were isolated using the quadrupole analyzer in a window of 1.6 m/z and fragmented by higher energy collision-induced dissociation (HCD) with 35% of collision energy. The resulting fragments were detected by the linear ion trap at a rapid scan rate with an AGC target of 1 × 10^4^ and a maximum injection time of 50 ms. The dynamic exclusion of previously fragmented peptides was set for a period of 30 s and a tolerance of 10 ppm.

### 4.6. Quantitative Data Analyses

Spectra were searched against the Uniprot Reference mus musculus (61,295 entries) using the Andromeda module of the MaxQuant software, v. 1.6.10.43. The trypsin/P enzyme parameter was selected with two possible missed cleavages. The carbamidomethylation of cysteines was set as a fixed modification, while methionine oxidation and phosphorylation (Ser, Thr, Tyr) were set as variable modifications. The mass search tolerances were 5 ppm and 0.5 Da for MS and MS/MS, respectively. For the protein validation, a maximum false discovery rate of 1% at the peptide and protein levels was used based on a target/decoy search. MaxQuant was also used for label-free quantifications. The ‘match between runs’ option was used with a 20 min value as the alignment time window and 0.7 min as the match time window. For the global protein analysis, the protein group files were used, while for the phosphopeptide analysis, the modificationSpecifPeptides file was used. RStudio 1.2.5019 was used for data processing. A normalization step was performed using the median of the median intensities of each condition. The missing peptide intensity values were replaced by a noise value corresponding to the 1% percentile of the normalized value for each condition. A peptide was considered quantifiable only if at least three intensity values in one of the two conditions were present and with a minimum of two peptides (for the global protein analysis).

### 4.7. Statistical Analysis

For the RT-qPCR, statistical analyses were carried out using a nonparametric one-way ANOVA on ranks via the Kruskal–Wallis test, followed by a post-hoc Mann–Whitney U test. A *p*-value of < 0.05 was considered significant. For the LC-MS/MS analysis, a *p*-value limma test, a limma *q*-value (Benjamin–Hochberg correction), and a z-score were calculated. Phosphopeptides or proteins were considered variants if the *q*-value was < 0.05 and the z-score was +/− 1.96. All statistical analyses were performed using the OriginPro Version 2021 software (www.originlab.com, accessed on 10 August 2022) (OriginLab Corporation, Northampton, MA, USA).

## Figures and Tables

**Table 1:** Variation in protein levels from MA-10 Leydig cells treated for 1 h with vehicle (DMSO), Fsk, or Fsk+AICAR.

DMSO vs. Fsk							
***Upregulated by Forskolin***							
Protein name	Protein	Gene name	Fsk	DMSO	Fsk/DMSO	*q*-Value	Fsk/DMSO
Dymeclin	Q8CHY3	*Dym*	3.64 × 10^7^	1.57 × 10^6^	23.2	5.08 × 10^-6^	Up
Homologous-pairing protein 2 homolog	O35047	*Psmc3ip*	7.93 × 10^6^	1.57 × 10^6^	5.06	1.07 × 10^-4^	Up
Acyl-Coenzyme A dehydrogenase family member 12	D3Z7X0	*Acad12*	8.76 × 10^6^	2.22 × 10^6^	3.94	2.56 × 10^-2^	Up
N-acetyltransferase domain containing 1	Q9DBW3	*Natd1*	5.61 × 10^6^	1.57 × 10^6^	3.58	1.58 × 10^-4^	Up
Nuclear receptor subfamily 4 group A member 1	P12813	*Nr4a1*	3.76 × 10^6^	1.57 × 10^6^	2.4	1.41 × 10^-3^	Up
***Downregulated by Forskolin***							
Protein name	Protein	Gene name	Fsk	DMSO	DMSO/Fsk	*q*-Value	Fsk/DMSO
H/ACA ribonucleoprotein complex subunit 3	Q9CQS2	*Nop10*	1.21 × 10^6^	7.91 × 10^6^	6.55	1.44 × 10^-2^	Down
Mpv17-like protein 2	Q8VIK2	*Mpv17l2*	1.21 × 10^6^	6.46 × 10^6^	5.35	8.13 × 10^-3^	Down
Overexpressed in colon carcinoma 1 protein homolog	P0C913	*Occ1*	1.21 × 10^6^	6.36 × 10^6^	5.27	8.32 × 10^-3^	Down
Glutathione peroxidase 7	Q99LJ6	*Gpx7*	1.21 × 10^6^	4.79 × 10^6^	3.97	3.60 × 10^-2^	Down
**Fsk vs. Fsk+AICAR**							
***Upregulated by AICAR***							
Protein name	Protein	Gene name	Fsk+AICAR	Fsk	Fsk+AICAR/Fsk	*q*-Value	Fsk+AICAR/Fsk
Cyclic AMP-responsive element-binding protein 1	Q01147	*Creb1*	2.19 × 10^7^	1.21 × 10^6^	18.15	3.83 × 10^-4^	Up
Mpv17-like protein 2	Q8VIK2	*Mpv17l2*	6.32 × 10^6^	1.21 × 10^6^	5.23	5.72 × 10^-3^	Up
Overexpressed in colon carcinoma 1 protein homolog	P0C913	*Occ1*	5.95 × 10^6^	1.21 × 10^6^	4.93	7.06 × 10^-3^	Up
HAUS augmin-like complex subunit 3	Q8QZX2	*Haus3*	3.98 × 10^6^	1.21 × 10^6^	3.3	3.10 × 10^-2^	Up
***Downregulated by AICAR***							
Protein name	Protein	Gene name	Fsk+AICAR	Fsk	Fsk/Fsk+AICAR	*q*-Value	Fsk+AICAR/Fsk
Dymeclin	Q8CHY3	*Dym*	1.61 × 10^6^	3.64 × 10^7^	22.64	8.11x 10^-6^	Down
Ubinuclein-2	Q80WC1	*Ubn2*	1.61 × 10^6^	1.56 × 10^7^	9.7	1.71 × 10^-5^	Down
Ferrochelatase	P22315	*Fech*	1.61 × 10^6^	6.69 × 10^6^	4.16	4.02 × 10^-4^	Down
Serine/threonine-protein kinase LMTK2	Q3TYD6	*Lmtk2*	1.61 × 10^6^	4.56 × 10^6^	2.84	6.67 × 10^-3^	Down
Armadillo-like helical domain-containing protein 3	Q6PD19	*Armh3*	1.61 × 10^6^	4.52 × 10^6^	2.81	8.76 × 10^-4^	Down
Ribokinase	Q8R1Q9	*Rbks*	1.61 × 10^6^	4.05 × 10^6^	2.52	1.13 × 10^-3^	Down
Nuclear receptor subfamily 4 group A member 1	P12813	*Nr4a1*	1.61 × 10^6^	3.76 × 10^6^	2.34	1.48 × 10^-3^	Down
**DMSO vs. Fsk+AICAR**							
***Upregulated by Forskolin + AICAR***							
Protein name	Protein	Gene name	Fsk+AICAR	DMSO	Fsk+AICAR/DMSO	*q*-Value	Fsk+AICAR/DMSO
Glutamyl-tRNA(Gln) amidotransferase subunit B. mitochondrial	Q99JT1	*Gatb*	3.56 × 10^6^	1.57 × 10^6^	2.27	8.86 × 10^-4^	Up
Decapping and exoribonuclease protein	O70348	*Dxo*	2.89 × 10^6^	1.57 × 10^6^	1.84	3.70 × 10^-2^	Up
***Downregulated by Forskolin + AICAR***							
Protein name	Protein	Gene name	Fsk+AICAR	DMSO	DMSO/Fsk+AICAR	*q*-Value	Fsk+AICAR/DMSO
SAGA-associated factor 29	Q9DA08	*Sgf29*	1.61 × 10^6^	1.39 × 10^9^	864.22	3.00 × 10^-5^	Down
Coiled-coil domain-containing protein 187	Q8C5V8	*Ccdc187*	1.61 × 10^6^	1.40 × 10^7^	8.71	3.87 × 10^-4^	Down
Ferrochelatase	P22315	*Fech*	1.61 × 10^6^	6.73 × 10^6^	4.19	1.34 × 10^-4^	Down
Armadillo-like helical domain-containing protein 3	Q6PD19	*Armh3*	1.61 × 10^6^	5.59 × 10^6^	3.48	3.67 × 10^-2^	Down

**Table 2:** Top 10 phosphopeptides differentially phosphorylated in MA-10 Leydig cells treated with vehicle (DMS), Fsk, or Fsk+AICAR (pair comparisons).

Fsk vs. DMSO								
***Phosphorylation decreased by Forskolin***								
Phosphopeptide Sequence	Protein	Protein Name	Gene Name	Fsk	DMSO	DMSO/ Fsk	*q*-Value	Fsk/ DMSO
IQTSNVTNKNDPK	Q64012	RNA-binding protein Raly	*Raly*	2.12 × 10^6^	3.47 × 10^8^	163.6	4.54 × 10^-4^	Down
DLPPFEDESEGLLGTEGPMEEEEDGEELIGDGMER	P97310	DNA replication licensing factor MCM2	*Mcm2*	2.12 × 10^6^	5.46 × 10^7^	25.7	1.63 × 10^-3^	Down
IDEPNTPYHNMIGDDEDAYSDSEGNEVMTPDILAK	Q9DCL8	Protein phosphatase inhibitor 2	*Ppp1r2*	2.12 × 10^6^	5.14 × 10^7^	24.19	1.53 × 10^-3^	Down
HLSNVSSTGSIDMVDSPQLATLADEVSASLAK	P10637	Microtubule-associated protein tau	*Mapt*	2.12 × 10^6^	4.06 × 10^7^	19.14	1.53 × 10^-3^	Down
SSSPSASLTEHEVSDSPGDEPSESPYESADETQTEASVSSK	Q8BVE8	Histone-lysine N-methyltransferase NSD2	*Whsc1*	2.12 × 10^6^	3.51 × 10^7^	16.54	8.63 × 10^-3^	Down
ETGGTYPPSPPPHSSPTPAATVAATVSTAVPGEPLLPR	Q80U72	Protein scribble homolog	*Scrib*	2.12 × 10^6^	2.36 × 10^7^	11.13	1.55 × 10^-2^	Down
RISHSLYSGIEGLDESPTR	Q99NH2	Partitioning defective 3 homolog	*Pard3*	2.12 × 10^6^	2.19 × 10^7^	10.33	2.37 × 10^-2^	Down
KSMYSRVPECQVTTYYYVGFAYLMMR	Q8QZY1	Eukaryotic translation initiation factor 3 subunit L	*Eif3l*	2.12 × 10^6^	2.12 × 10^7^	9.99	3.82 × 10^-3^	Down
ALEETPPDSPAAEQENSVNCVDPLR	Q8K1K3	TERF1-interacting nuclear factor 2	*Tinf2*	2.12 × 10^6^	2.03 × 10^7^	9.54	3.36 × 10^-3^	Down
LAAQESSEAEDVTVDR	Q6PGL7	WASH complex subunit FAM21	*Fam21*	2.12 × 10^6^	1.99 × 10^7^	9.36	4.73 × 10^-2^	Down
***Phosphorylation increased by Forskolin***								
Phosphopeptide Sequence	Protein	Protein Name	Gene Name	Fsk	DMSO	Fsk/ DMSO	*q*-Value	Fsk/ DMSO
KSSVEGLEPAENK	P16254	Signal recognition particle 14 kDa protein	*Srp14*	2.63 × 10^8^	2.90 × 10^6^	90.6	4.54 × 10^-4^	Up
STSQGSINSPVYSR	Q8K4G5	Actin-binding LIM protein 1	*Ablim1*	7.08 × 10^7^	2.12 × 10^6^	33.48	1.63 × 10^-3^	Up
QNPEQSADEDAEKNEEDSEGSSDEDEDEDGVGNTTFLK	Q8R1B4	Eukaryotic translation initiation factor 3 subunit C	*Eif3c*	7.06 × 10^7^	2.12 × 10^6^	33.39	1.53 × 10^-3^	Up
PCIQAQYGTPATSPGPR	P12813	Nuclear receptor subfamily 4 group A member 1	*Nr4a1*	4.52 × 10^7^	2.12 × 10^6^	21.36	1.53 × 10^-3^	Up
NTFTAWSEEDSDYEIDDR	Q6ZQ58	La-related protein 1	*Larp1*	1.66 × 10^8^	1.02 × 10^7^	16.28	9.61 × 10^-3^	Up
EKVESAGPGGDSEPTGSTGALAHTPR	O08550	Histone-lysine N-methyltransferase 2B	*Kmt2b*	2.42 × 10^7^	2.12 × 10^6^	11.44	3.17 × 10^-3^	Up
SPDLSNQNSDQANEEWETASESSDFASER	Q7TSC1	Protein PRRC2A	*Prrc2a*	1.77 × 10^7^	2.12 × 10^6^	8.35	8.52 × 10^-3^	Up
KVSVEPQDSHQDAQPR	Q8BXB6	Solute carrier organic anion transporter family member 2B1	*Slco2b1*	1.97 × 10^8^	2.47 × 10^7^	7.98	1.30 × 10^-2^	Up
KSSAAAAAAAAAEGALLPQTPPSPR	Q9ERD6	Ras-specific guanine nucleotide-releasing factor RalGPS2	*Ralgps2*	1.64 × 10^7^	2.12 × 10^6^	7.77	9.61 × 10^-3^	Up
SHSESASPSALSSSPNNLSPTGWSQPK	Q99N57	RAF proto-oncogene serine/threonine-protein kinase	*Raf1*	1.61 × 10^7^	2.12 × 10^6^	7.63	1.30 × 10^-2^	Up
**Fsk+AICAR vs. Fsk**								
***Phosphorylation decreased by AICAR***								
Phosphopeptide Sequence	Protein	Protein Name	Gene Name	Fsk + AICAR	Fsk	Fsk/ Fsk+AICAR	*q*-Value	Fsk+AICAR/Fsk
YDEKTGLALQTEEFIPNYYCTDERR	K7N6T2	Vomeronasal 2 receptor 67	*Vmn2r67*	2.15 × 10^6^	1.56 × 10^9^	727.23	1.81 × 10^-4^	Down
GASAATGIPLESDEDSNDNDNDLENENCMHTN	Q8R5H1	Ubiquitin carboxyl-terminal hydrolase 15	*Usp15*	2.15 × 10^6^	6.62 × 10^7^	30.83	2.50 × 10^-3^	Down
KGSDDDGGDSPVQDIDTPEVDLYQLQVNTLR	O88574	Histone deacetylase complex subunit SAP30	*Sap30*	2.15 × 10^6^	3.75 × 10^7^	17.43	1.12 × 10^-2^	Down
WGQPPSPTPVPRPPDADPNTPSPK	Q6PDQ2	Chromodomain-helicase-DNA-binding protein 4	*Chd4*	2.15 × 10^6^	3.33 × 10^7^	15.52	8.64 × 10^-3^	Down
TNSMGSATGPLPGTK	Q8BZ47	Zinc finger protein 609	*Znf609*	2.15 × 10^6^	3.21 × 10^7^	14.94	4.99 × 10^-3^	Down
NLATSADTPPSTIPGTGK	Q8BKX6	Serine/threonine-protein kinase SMG1	*Smg1*	2.15 × 10^6^	1.63 × 10^7^	7.57	4.56 × 10^-3^	Down
SPHDSKSPLDHRSPLER	E9PZM4	Chromodomain-helicase-DNA-binding protein 2	*Chd2*	2.15 × 10^6^	1.58 × 10^7^	7.35	4.63 × 10^-3^	Down
IASSSSENNFLSGSPSSPMGDILQTPQFQMR	E9Q7G0	Nuclear mitotic apparatus protein 1	*Numa1*	2.15 × 10^6^	1.40 × 10^7^	6.52	1.14 × 10^-2^	Down
NQENVSHLSVSSASPTSSVASAAGSVTSSSLQK	O54826	Protein AF-10	*Mllt10*	2.15 × 10^6^	1.39 × 10^7^	6.45	2.00 × 10^-2^	Down
GSSGEGLPFAEEGNLTIK	Q8VHK1	Caskin-2	*Caskin2*	2.15 × 10^6^	1.23 × 10^7^	5.75	1.02 × 10^-2^	Down
***Phosphorylation increased by AICAR***								
Phosphopeptide Sequence	Protein	Protein Name	Gene Name	Fsk + AICAR	Fsk	Fsk+AICAR/ Fsk	*q*-Value	Fsk+AICAR/Fsk
ARPAQAPVSEELPPSPKPGK	Q6PGL7	WASH complex subunit FAM21	*Fam21*	6.22 × 10^7^	2.12 × 10^6^	29.29	1.54 × 10^-2^	Up
HLSNVSSTGSIDMVDSPQLATLADEVSASLAK	P10637	Microtubule-associated protein tau	*Mapt*	4.88 × 10^7^	2.12 × 10^6^	22.99	2.50 × 10^-3^	Up
APSPEPPTEEVAAETNSTPDDLEAQDALSPETTEEK	Q9ESJ4	NCK-interacting protein with SH3 domain	*Nckipsd*	4.73 × 10^7^	2.12 × 10^6^	22.26	2.50 × 10^-3^	Up
SSSPSASLTEHEVSDSPGDEPSESPYESADETQTEASVSSK	Q8BVE8	Histone-lysine N-methyltransferase NSD2	*Whsc1*	3.40 × 10^7^	2.12 × 10^6^	16.00	4.63 × 10^-3^	Up
VLLTHEVMCSR	O08792	Transcription factor COE2	*Ebf2*	3.04 × 10^7^	2.12 × 10^6^	14.33	2.50 × 10^-3^	Up
VTETEDDSDSDSDDDEDDVHVTIGDIK	Q9D824	Pre-mRNA 3’-end-processing factor FIP1	*Fip1l1*	2.40 × 10^7^	2.12 × 10^6^	11.31	2.50 × 10^-3^	Up
SRSVEDDEEGHLICQSGDVLSAR	P22518	Dual specificity protein kinase CLK1	*Clk1*	2.04 × 10^7^	2.12 × 10^6^	9.63	8.25 × 10^-3^	Up
RISHSLYSGIEGLDESPTR	Q99NH2	Partitioning defective 3 homolog	*Pard3*	1.94 × 10^7^	2.12 × 10^6^	9.16	3.87 × 10^-3^	Up
DAEDLSPCLPSSSQEDTAVPSSPGPSDEVSNTEAEAR	G5E8P0	Gamma-tubulin complex component 6	*Tubgcp6*	1.92 × 10^7^	2.12 × 10^6^	9.06	3.47 × 10^-2^	Up
SVDLKTASPESGRSGFQDEESFR	E9Q4Y4	Centrosomal protein 192	*Cep192*	1.89 × 10^7^	2.12 × 10^6^	8.91	4.99 × 10^-3^	Up
**Fsk+AICAR vs. DMSO**								
***Phosphorylation decreased by Forskolin + AICAR***								
Phosphopeptide Sequence	Protein	Protein Name	Gene Name	Fsk + AICAR	DMSO	DMSO/Fsk+AICAR	*q*-Value	Fsk+AICAR/DMSO
AADPPAENSSAPEAEQGGAE	P62960	Nuclease-sensitive element-binding protein 1	*Ybx1*	2.15 × 10^6^	1.60 × 10^8^	74.33	2.97 × 10^-3^	Down
DLPPFEDESEGLLGTEGPMEEEEDGEELIGDGMER	P97310	DNA replication licensing factor MCM2	*Mcm2*	2.15 × 10^6^	5.46 × 10^7^	25.4	2.27 × 10^-3^	Down
SLTISVDSASTSR	Q99MZ6	Unconventional myosin-VIIb	*Myo7b*	2.15 × 10^6^	4.54 × 10^7^	21.12	3.65 × 10^-2^	Down
LDGESDKEQFDDDQK	Q8C1B1	Calmodulin-regulated spectrin-associated protein 2	*Camsap2*	2.15 × 10^6^	3.27 × 10^7^	15.23	6.41 × 10^-3^	Down
ALEETPPDSPAAEQENSVNCVDPLR	Q8K1K3	TERF1-interacting nuclear factor 2	*Tinf2*	2.15 × 10^6^	2.03 × 10^7^	9.43	4.79 × 10^-3^	Down
QATESPAYGIPLKDGSEQTDEEAEGPFSDDEMVTHK	Q99KK1	Receptor expression-enhancing protein 3	*Reep3*	2.15 × 10^6^	1.89 × 10^7^	8.8	2.18 × 10^-2^	Down
HSPIKEEPCGSISETVCK	Q6PDM1	Male-specific lethal 1 homolog	*Msl1*	2.15 × 10^6^	1.88 × 10^7^	8.75	2.63 × 10^-2^	Down
QDVETCRPSSPFGR	Q6PG16	Holliday junction recognition protein	*Hjurp*	2.15 × 10^6^	1.78 × 10^7^	8.27	1.96 × 10^-2^	Down
SSPDPAVNPVPK	Q924W7	Suppression of tumorigenicity 5 protein	*St5*	2.15 × 10^6^	1.73 × 10^7^	8.03	6.41 × 10^-3^	Down
SEVNSEDSDIQEVLPVPK	Q7TQI8	Testis-specific Y-encoded-like protein 2	*Tspyl2*	2.83 × 10^6^	2.20 × 10^7^	7.8	1.83 × 10^-2^	Down
***Phosphorylation increased by Forskolin + AICAR***								
Sequence	Protein	Protein Name	Gene Name	Fsk + AICAR	DMSO	Fsk+AICAR/ DMSO	*q*-Value	Fsk+AICAR/DMSO
KSSVEGLEPAENK	P16254	Signal recognition particle 14 kDa protein	*Srp14*	2.76 × 10^8^	2.90 × 10^6^	95.06	1.47 × 10^-3^	Up
TPLGASLDEQSSGTPK	Q922B9	Sperm-specific antigen 2 homolog	*Ssfa2*	1.37 × 10^8^	2.35 × 10^6^	58.37	1.47 × 10^-3^	Up
TVSTQHSTESQDNDQPDYDSVASDEDTDVETR	Q9JLQ2	ARF GTPase-activating protein GIT2	*Git2*	1.84 × 10^8^	3.32 × 10^6^	55.33	6.87 × 10^-3^	Up
QNPEQSADEDAEKNEEDSEGSSDEDEDEDGVGNTTFLK	Q8R1B4	Eukaryotic translation initiation factor 3 subunit C	*Eif3c*	8.66 × 10^7^	2.12 × 10^6^	40.93	1.74 × 10^-3^	Up
STSQGSINSPVYSR	Q8K4G5	Actin-binding LIM protein 1	*Ablim1*	8.20 × 10^7^	2.12 × 10^6^	38.77	3.41 × 10^-3^	Up
LKNDSDLFGLGLEEMGPKESSDEDR	Q5U3K5	Rab-like protein 6	*Rabl6*	4.16 × 10^7^	2.12 × 10^6^	19.67	1.76 × 10^-3^	Up
NTFTAWSEEDSDYEIDDR	Q6ZQ58	La-related protein 1	*Larp1*	1.88 × 10^8^	1.02 × 10^7^	18.39	6.41 × 10^-3^	Up
AELEEMEEVHPSDEEEEETKAESFYQK	Q9QYR6	Microtubule-associated protein 1A	*Map1a*	3.86 × 10^7^	2.12 × 10^6^	18.26	1.76 × 10^-3^	Up
QSNASSDVEVEEKETNVSKEDTDQEEK	Q99JF8	PC4 and SFRS1-interacting protein	*Psip1*	3.81 × 10^7^	2.12 × 10^6^	18.01	6.41 × 10^-3^	Up
PCIQAQYGTPATSPGPR	P12813	Nuclear receptor subfamily 4 group A member 1	*Nr4a1*	2.46 × 10^7^	2.12 × 10^6^	11.62	2.39 × 10^-2^	Up

## Data Availability

All mass spectrometry data (raw files and MaxQuant search result files) are publicly available on the ProteomeXchange repository (www.proteomexchange.org) with the identifier PXD037514.
